# Editorial: Neurocardiovascular Diseases: New Aspects of the Old Issues

**DOI:** 10.3389/fnins.2018.01032

**Published:** 2019-01-11

**Authors:** Tijana Bojić

**Affiliations:** Laboratory of Radiobiology and Molecular Genetics, Institute of Nuclear Sciences Vinča, University of Belgrade, Belgrade, Serbia

**Keywords:** neurocardiovascular diseases, Integrative pathology, autonomic nervous system, sympathetic, parasympathetic, development

Concept of neuro-cardiovascular diseases (NCVD) is one fruitful approach that enables the comprehension of the pathological processes raising along the brain-heart & blood vessel axes from the integrative prospective. That is the reason why multidisciplinary and interdisciplinary approaches result in novel insights into pathophysiological processes bringing new directions for therapeutic development, both at the laboratory bench and at the clinical bedside. NCVD comprises the group of pathologies that have as a primary pathological substrate the changes in neurochemical, neurophysiological and neuroanatomical levels of the autonomic nervous system (ANS) and its regulated organs (e.g., heart, blood vessels).

A striking fact is that “cardiovascular disease is the leading cause of death in the world today and will remain so by the year 2020” (The WHO MONICA Project, Investigators, 1988) strongly supports the need for new insights into cardiovascular regulatory mechanisms (Bojić, 2003). A picture of the classical cardiovascular risk factors from the prospective of neural cardiovascular control, links these factors with stress. The central topic of NCV physiology is related to stress-induced disfunction (e.g., hypertension, Du et al., 2017), emotional stress coping-cigarette smoking, obesity, Strickland et al., 2007) and stress-releasing strategies (exercise, Acevedo et al., 2006; Webb et al., 2017).

Central questions of neurophysiology of stress is:

- Identification of different brain networks activated during stress
- Spacial and temporal patterns of their activation
- Identification of neuronal hubs coupling cognitive-emotional neural networks with body effectors, like the hypothalamus-pituitary-adrenal (HPA) and the sympathetic-adrenomedullary axis (SAM) (Ulrich-Lai and Herman, 2009; Godoy et al., 2018).

The interaction of the heart and vessels with the central and peripheral nervous systems represents the major topic of the basic neuro-cardiovascular research, with the current aim of the field being to highlight the mechanisms of devastating effects of stress upon the cardiovascular system. In the review of Dampney the focus was on anatomical basis and functional role of the dorsolateral periaqueductal gray (dlPAG) in generating behavioral and autonomic responses to real and perceived emotional stressors. Central position and integratory function of this structure, between higher cortical regions (auditory, secondary visual, olphactory; medial prefrontal cortex -MPC, hypothalamus and lower brainstem structures), qualifies it as a crucial, emotional stress mediator neural hub. Though the review reports, parallel and comparative results obtained in different species, the guiding theme is the enlightening of the understanding of the morpho-functional substrate affiliated with emotional stress response in humans. Crucial for understanding of the behavioral response to the perceived emotional stress are the inputs from the MPC to the dlPAG. The species-specific MPC subregion of the primate brain, the area 10 m, has the largest volume in humans with respect to other primate species. Allman and collaborates (Allman et al., 2002) describe this structure as a comparator of current and memorized behavioral states, and as a consequent decision-maker about future, potentially advantageous behavior. Its high activation and direct interface with dlPAG points to its special role in complex emotional responses, resulting from such comparisons. In conclusion, dlPAG is presented as integrator of the reactions of both the conscious and the unconscious to threatening stimuli with dependent autonomic networks (i.e., cardiorespiratory network) which support the behavioral response to stress and threatening stimuli. Future studies need to address the questions of chemical phenotyping of the dlPAG and other extensive stress mediating brain networks; the question of morpho-functional plasticity with respect to timing and duration of stress exposure; association of stress-induced morpho-functional changes of critical brain networks associated with different cardiovascular pathologies, as well as the question of genetic predisposition to developing of specific pathological entities like NCVD on the order of short or long time scales.

The impact of peripheral information on neural mediation of the cardiovascular (CVS) response can be crucial to the development of pathologies initiated by physical stressors like injury. When a physical stressor is recognized by the brainstem through pain, inflammation and other signals, both fast SAM and sluggish HPA responses are activated (Godoy et al., 2018). Up to the present it is not known what the role of pre-stimulus of the ANS regime is for development of the compartment syndrome. In this line, the study that investigated the effect of peripheral neural input to the heart rate regulating network by Watanabe and Hotta for the first time examined the specific cardiac autonomic changes induced by the bio-mechanical pressure stimulation of skeletal muscle. The authors identified sympathetic nervous system as the effector of changes in heart rate and blood pressure. In addition, it was demonstrated that the tonic level of pre-stimulus sympathetic neural activity determines the direction of induced heart rate changes and changes to blood pressure. This data could be of particular importance for understanding the compartment syndrome, the condition of muscle ischemic necrosis due to excessive intramuscular pressure and blood hypoperfusion. Future studies are necessary for revealing the site of the interaction of peripheral muscle pressure, stretching and contraction stimulus of the CVS neural networks (spinal or brainstem), and chemical phenotyping for the purpose of pharmacological intervention, and the role of tonic pre-stimulus sympathetic neural activity, for the development of the hemodynamic profiles that are susceptible to progression of the compartment syndrome.

Drug targeting of spinal and brainstem autonomic neural circuits causally involved in the genesis of different NCVD could result with long-awaited pharmacological solutions (Pierce et al., 2010; Zimmerman, 2011) for unexpected groups of pathophysiological entities, like compartment syndrome and neurogenic hypertension.

Neurogenic hypertension (NH) and vasovagal syncope (VVS) are NCV entities representing an unsolved pathophysiological puzzle. Novel data exists about the central molecular mechanisms regulating the tonic activity of preganglionic sympathetic neurons (Zimmerman, 2011), with antagonizing effects of the angiotensin II receptor and the MAS1 receptor mediated cascade offer a promising perspective for *in silico* strategies for investigation of NH. The Information Spectrum Method (ISM), a virtual spectroscopy method for studying the long-range interactions between biological macromolecules (Veljković et al., 1985), was previously successfully applied in study of HIV (Veljković et al., 2007), anthrax (Doliana et al., 2008) and the influenza virus (Perović et al., 2013). This widely accepted method (Veljković et al., 2011) was applied for the first time by Bojić et al. for the investigation of molecular targets of NH and VVS. As the result of this study, there have been proposed three novel therapeutic candidates for treatment of NH (apelin-28, apelin-31 and apelin-36) and also 12 repurposed antimuscarinic drugs potentially could be efficient in VVS treatment. Follow up with *in vitro* and *in vivo* studies will test the therapeutic capacities of drug candidates identified by ISM.

Baroreflex sensitivity (BRS) represents one of the central research topics of NCV physiology in the last few decades (Bojić, 2003; Silvani et al., 2003, 2005; Zoccoli et al., 2005; Bajić et al., 2010; Kapidžić et al., 2014; Platiša et al.) and pathophysiology (Parati et al., 2004; Glišić et al., 2016). BRS has been recognized as valuable prognostic factor for the outcome of different NCVD like myocardial infarction (La Rovere et al., 1998), heart failure (Libbus et al., 2016) and hypertension (Subha et al., 2016). The methodology of BRS estimation evolved from the classical methods of BRS estimation based on induced blood pressure changes (mechanical, pharmacological, etc.) with the related research activity achieving considerable levels since the late 1950's (Ernsting and Parry, 1957; Lamberti et al., 1968; Kirchheim, 1976), up to the BRS techniques for analysis of blood pressure and HP spontaneous fluctuations, which were introduced during the late 1980's (Fritsch et al., 1986; Bojić, 2003).

Major advantages of spontaneous fluctuations method are:

1. There is no administration of vasoactive compounds or external appliances that could influence the baroreceptor reflex by a direct action on receptor or effector sites (Coleman, 1980).
2. BRS is measured within physiological ABP ranges, allowing the computation of the gain at ABP close to the operating set point value, with minimal nonspecific effects from other efferent nerves.
3. The method does not arouse subjects or animals, thereby reducing stress induced effects.
4. In contrast with pharmacological or mechanical methods, they are suitable to assess the BRS over prolonged periods of time (Mancia and Mark, 1983; Oosting et al., 1997).

Still, time and frequency domain analysis of ANS activity in cardiovascular signals require certain conditions, with a stable baseline as one of the most important and most difficult states to obtain on long data segments. Li et al. propose a Multiple Trigonometric Regressive Spectral Analysis as a novel method for baroreflex sensibility (BRS) estimation for short (20–30's) time segments. The proposed method uses the oscillations of ABP and HP instead of their original values. The method provides reliable estimates of BRS without regard to posture change during the short data segments of 20–30 s in length. The proposed method solves several shortcomings of the sequence method by increasing the accuracy and validity of BRS estimation (Ziemssen et al., 2013), by providing a pure physiological spectrum of ABP and HP fluctuations and by reducing the influence of non-baroreflex drives. Further studies are necessary for evaluation of this promising method for studying BRS and other CVS indexes during the dynamic processes of daily life.

Atrial fibrillation (AF) presents NCVD events typically triggered by sympathovagal discharge (Goldstein, 2001), resulting in a dysfunctional atrial rhythm possessing as a consequence stroke, heart failure and risk of dementia. The electrical instability of atria, both focal and re-entrant activity, are progressive, self-feedback processes that evolve paroxysmal AF toward its persistent form. These classical experimental and clinical observations were without clear evidence-based pathophysiological explanation (Schotten et al., 2011). Ashton et al. propose the morpho-functional remodeling of ANS, both of its extrinsic (pre and post-ganglionic neurons) and intrinsic components (ganglionated plexus) that innervate the heart, as fundamentally contributing to positive feedback mechanism of AF. The ganglionated plexus, is the network of acetylcholine and other neurochemically distinct neurons, which play an important role in the modulation of cholinergic transmission and can be the site of maladaptive changes including arrhythmogenesis. These maladaptive changes could be based on both short-term and long-term plasticity mechanisms, with engagement of 5HT_3_ receptors, acetylcholine release and NO signaling in sympathetic neurons, and nicotinic expression, NO-cGMP signaling and NMDA receptor expression in vagal neurons. The neurochemical profile of synaptic plasticity of both sympathetic and vagal ganglionic transmission is promising target for future pharmacological studies aiming to intervene in AF that is morpho-functionally stabile as its persistent form. Traditional ECG and HRV linear and nonlinear indexes (Kikillus et al., 2007) could not give the answers about regulatory mechanisms of longer time scales and their differences between healthy and AF patients. Differences in nonlinear HP functional patterns between healthy and AF patients could be of major importance for the diagnosis and consequent therapy of AF forms that are of central neural origin (Andrade et al., 2014).

This was the focus of the research of Platiša et al., where novel Generalized Poincaré Plot (GPP) analysis of RR interval was proposed as a sensible method for distinction of AF from healthy subjects. GPP revealed for the first-time different system dynamics for large time scales in AF and healthy subjects. In the special case when GPP analysis was performed between 100 preceding and 100 following RR intervals, distinct regimes could be observed in healthy subjects, reflecting hypothetical different set-points of the blood pressure-heart rate baroreflex loop. In AF patients the GPP profile of RR intervals were scattered. This result suggests that AF patients have smaller adaptive capacity to internal and external perturbations. Four cluster profile of correlation maxima and their absolute values for different correlation scales were also different in healthy and AF patients. These results supported the hypothesis that regulatory regimes in healthy subjects operate in fine tuned superimposed regimes acting on different time scales-parasympathetic, sympathetic and slow regulatory mechanisms like thermoregulation, rennin-angiotensin-aldosterone-sodium system, hormones etc. The AF cluster pattern was highly distorted, shifted toward higher frequencies and with increased randomness. The new GPP methodological approach for detection and profiling cardiovascular regimes need future pharmacological evaluation and potential translatory development as a diagnostic tool for AF and other NCVD.

ANS is coupled directly to the cardiovascular system, but also through the interface of an energy regulating system. This is why an imbalance of energy regulation, as it is the case in obesity, often represents the first step, or the initial trigger of a neurally and metabolically mediated cascade of cardiovascular complications (hypertension, generalized atherosclerotic diathesis, dyslipidemia, diabetes mellitus type II). Digestion, absorption and neuroendocrine activity associated with the adoption of food has a direct effect on cardiovascular regulating centers (de Lartigue, 2014), pointing to the vagal subsystem as the potential target for neuromodulatory and pharmacological interventions in treatment of obesity, and, consequently, obesity related diseases. This important aspect of NCVD was reviewed by Guarino et al. Even though this comprehensive overview emphasizes the potential vagal route for neuromodulation and pharmacological intervention, the sympathetic route was also evaluated. This, more complex and differently structured subsystem presents itself as less understood and consequently is a significantly diminished path for obesity and related NCVD treatment. Its anatomical characteristics, i.e., approachability by external manipulations imply that the pharmacological approach should be investigated in the future, while the vagal route offers a good basis for both neuromodulatory and pharmacological strategies. Vagal modulation, in specific transcutaneous auricular vagus nerve stimulation is a promising method for body weight reduction in obese patients, also resulting with significant improvements of cardiometabolic profile. Sympathetic modulation, with inconsistent results on body weight reduction and partial cardiometabolic effects, from a results prospective, is a less promising strategy.

Hyperadrenergic state is the classical hallmark of heart failure (HF), the clinical endpoint of the number of CVD (Marwick, 2018). Toschi-Dias et al. details elaborates the neurohumoral responses to hemodynamic stress, the common initial event of the HF hyperadrenergic state. The HF hyperadrenergic state, associated with different reductions of left ventricular ejection fraction (LVEF: preserved-p, mid-range-mr, reduced-r) is also associated with different morpho-functional remodeling of left ventricle, specific for its ejection functioning. This interesting association, widely recognized as a valuable prognostic and diagnostic parameter (Marwick, 2018) seeks for a deeper genetic and/or environmental influence studies, due to the hypothesis that different pathophysiological patterns might sculpture different EF phenotypes in HF. Vascular remodeling in HF further complicates an ANS functional profile, pushing it toward the maintenance and/or enhancement of hyperadrenergic state. Arterial baroreceptor (ABR) dysfunction, mostly due to the decrease of large vessel elasticity following chronic hypertension, distinguishes itself as an important factor in generation of cardiac diastolic dysfunction. ABR seem to play dominant role in sustaining hyperadrenergic state both in HFrEF (low stroke volume) and HFpEF (increased vascular stiffness), by different mechanisms. This issue necessitates future investigations of the hierarchical (in sense of absolute and relative quantitative contribution to the hyperadrenergic state) and temporal order of cardiovascular reflexes engaged in HFmrEF and HFpEF. It is reasonable to hypothesize that different quantitative and temporal patterns of cardiovascular reflex response result with different HFEF phenotypes. Cardiopulmonary reflex (CPR) regulates the state of systemic blood volume by (a) sympathetic modulation (low intensity changes), (b) release of atrial Na-uretic peptide, and (c) by strengthening and enhancing ABR action at high intensity changes. In HFrEF patients, no reduction of sympathetic outflow is obtained by CPR unloading. Participation of cardiac sympathetic afferent reflex and arterial chemoreflex was thoroughly evidenced in the hyperadrenergic state of HFrEF, while their role in HFpEF and HFmrEF needs future evaluation.

A number of surgical and pharmacological interventions manifest NCV disturbances as an important caveat. For that reason, detailed and comprehensive NCV evaluation becomes a constituent part of pre-interventional evaluation of the patient and postintervention follow up. Coronary artery bypass graft (CABG) surgery can induce disbalance of sympathovagal ratio and, consequently respiratory depression, approximately 5 days after the surgery (Aronson et al., 2011; Pantoni et al., 2014; Patron et al., 2014; Ksela et al., 2015). In order to identify a prognostic marker, Costa et al. studied the prognostic significance of perioperative arterial blood pressure (ABP) variability for the occurrence of respiratory depression following CABG. The finding of Costa et al. that ABP variability parameters have prognostic value for respiratory depression has both pathophysiological and clinical significance.

Deep brain stimulation (DBS) represents an invasive, frequent and developing intervention for the treatment of a spectrum of neurological diseases, with Parkinson's disease (PD) as the most common. As reported by Chowdhury et al., hemodynamic perturbations, like hypertension, hypotension, bradycardia, tachycardia and arrhythmia are frequent side-effects of this procedure in PD patients. They can be the consequence of (a) the independent or accompanying autonomic co-morbidity of the main PD pathological process, (b) the procedures associated to the surgery (semi-sitting position, anesthetics, sedation, stress, electrode battery placement and the stimulation of brain nuclei itself). Significant predictor potential has only pre-operative ABP, with diastolic BP as the marker most associated with hemodynamic event. Hypertension, predisposing factor of cerebral hemorrhage was noted during electrode placement and nuclei stimulation. A prospective study is needed for detailed hemodynamic evaluation (a) during the DBS surgery and the estimation of (b) pre- and (c) post-operative autonomic status of the PD patients. This approach would potentially change protocols of presurgical evaluation and postsurgical treatment of PD patients subjected to DBS. After heart transplantation, the autonomic reinnervation of the transplanted heart has important consequences on its reactivity and hemodynamical adaptability (exercise capacity, coronary blood flow regulation (Grupper et al., 2018). Wdowczyk et al. present a novel tool, Transition Networks, in the case report that has potential for distinguishing HRV increase due to reinnervation. Further stratified longitudinal clinical studies are needed for evaluation of this method. However, the capacity of the method to offer an insight into dynamical inter-beat dependences of RR intervals enounce better comprehension of the transplant functional adoption into CVS neural network.

Pharmacological interventions in neuropsychiatric patients often disturb autonomic balance. Li et.al. applied their method (long-term Multiple Trigonometric Spectral Analysis, Li et al.) combined with conventional liner parameters of HRV as a tool for predicting fingolimod-induced bradycardia in patients with multiple sclerosis (MS). On the basis of their analysis they report an increased pre medication parasympathetic activity as the predisposing factor for fingolimod induced bradycardia, with pretreatment HR as the only predicting factor. Yuen et al. performed the meta-analysis on clozapine induced autonomic dysfunction in patients with schizophrenia. They conclude that the most frequent complications were myocarditis, orthostatic hypotension and tachycardia, prevalently due to sympathetic overactivity. This report emphasizes the need for introduction of post-medication NCV evaluation autonomic tests for prevention and therapeutic coping with clozapine-induced side-effects. In accordance with Li et al., an intuitive direction for future investigations would be the identification of NCV predicting parameters for clozapine induced autonomic side effects.

Cerebral blood flow (CBF) is an issue of the upmost importance for understanding the NCVD, from two aspects:

- A growing corpus of data supports the standpoint that besides the cerebral autoregulation (Silvani et al., 2004; Zoccoli et al., 2005), sympathetic (Cassaglia et al., 2008; ter Laan et al., 2013; Frederiksen et al., 2017), parasympathetic (Purkayastha et al., 2018) and sensory innervations (Branston et al., 1995) functionally participate in the regulation of CBF.
- Compromise of CBF, especially in the neonates, can cause serious, life threatening autonomic dysfunctions (Silvani et al., 2004; Metzler et al., 2017; Campbell et al., 2018).

Glutamate is considered to be the neurochemical initiator and executor of brain injury in hypoxic-ischemic brain disorder (HIBD). Dang et al. report “two phase” change of basal ganglia glutamate level after HIBD, that is significantly and negatively correlated to the brain perfusion fraction. Even though this association is suggestive for negative electrochemical coupling of cerebral activity (glutamate) and brain perfusion in HIBD, further investigations are necessary for elucidating the mechanism(s) of brain activity-blood flow coupling in HIBD. Special emphasis should be on the role of sympathetic nervous system in cerebral activity-blood flow regulation in HIBD (Ainslie, 2008; Cassaglia et al., 2008; Edvinsson, 2008; Immink and Passier, 2008; Levine and Zhang, 2008; Ogoh, 2008; Paulson and Knudsen, 2008; Prakash, 2008; Visocchi, 2008; Yildiz, 2008; ter Laan et al., 2013). An important protective effect of artesunate against necrosis in cerebral infarction was reported by Shao et al., suggesting an autophagy as the most probable mechanism. Potential treatment by artesunate could have beneficiary effect for autonomic dysfunction, an important caveat of neonatal hypoxic-ischemic encephalopathy (Metzler et al., 2017; Campbell et al., 2018).

In conclusion, the presented physiological, methodological and pathophysiological aspects of NCVD point to the importance of consideration of NCVD from integrative point of view and as a constitutive part of different pathophysiological entities. Application of novel mathematical methods for molecular targeting and systemic characterizing of NCVD enounce promising lines of future research in the translational science. Neurocardiovascular side-effects of surgical and pharmacological interventions emphasize the importance of ANS evaluation before and after the intervention as the routine procedure in the clinical work. Finally, an intriguing role of autonomic networks, traditionally considered the cardiovascular neural subsystem, in the regulation of cerebral blood flow both in physiological and pathophysiological conditions is about to open a novel aspect of cerebro-cardiovascular integration.

## Author Contributions

The author confirms being the sole contributor of this work and has approved it for publication.

### Conflict of Interest Statement

The author declares that the research was conducted in the absence of any commercial or financial relationships that could be construed as a potential conflict of interest.
